# Association of hypertension severity and control with risk of incident atrial fibrillation: The REasons for Geographic And Racial Differences in Stroke (REGARDS) study

**DOI:** 10.1002/clc.24135

**Published:** 2023-08-22

**Authors:** Parveen K. Garg, Nicole Wilson, Ya Yuan, Virginia J. Howard, Suzanne Judd, George Howard, Elsayed Z. Soliman

**Affiliations:** ^1^ Division of Cardiology USC Keck School of Medicine Los Angeles California USA; ^2^ Department of Biostatistics University of Alabama at Birmingham Birmingham Alabama USA; ^3^ School of Public Health University of Alabama at Birmingham Birmingham Alabama USA; ^4^ Department of Epidemiology University of Alabama at Birmingham Birmingham Alabama USA; ^5^ Department of Medicine Epidemiological Cardiology Research Center (EPICARE), Section of Cardiovascular Medicine, Wake Forest School of Medicine Winston‐Salem North Carolina USA

**Keywords:** antihypertensive therapy, atrial fibrillation, hypertension, resistant hypertension

## Abstract

**Background:**

The association of hypertension (HTN) severity and control with the risk of incident atrial fibrillation (AF) is unclear.

**Hypothesis:**

Increased HTN severity and poorer blood pressure control would be associated with an increased risk of incident AF.

**Methods:**

This analysis included 9485 participants (mean age 63 ± 8 years; 56% women; 35% Black). Participants were stratified into six mutually exclusive groups at baseline—*normotension* (*n = 1629*), *prehypertension* (*n = 704*), *controlled HTN* (*n = 2224*), *uncontrolled HTN* (*n = 4123*), *controlled apparent treatment‐resistant hypertension (aTRH)* (*n = 88*), *and uncontrolled aTRH* (*n = 717*). Incident AF was ascertained at the follow‐up visit, defined by either electrocardiogram or self‐reported medical history of a physician diagnosis. Multivariable logistic regression analyses adjusted for demographic and clinical variables.

**Results:**

Over an average of 9.3 years later, 868 incident AF cases were detected. Compared to those with normotension, incident AF risk was highest for those with aTRH (controlled aTRH: odds ratio (OR) 2.95; 95% confidence interval (CI) 1.60, 5.43, & uncontrolled aTRH: OR 2.47; 95% CI 1.76, 3.48). The increase in AF risk was smaller for those on no more than three antihypertensive agents regardless of their blood pressure control (controlled OR 1.72; 95% CI 1.30, 2.29 and uncontrolled OR 1.56; 95% CI 1.14, 2.13).

**Conclusions:**

The risk of developing AF is increased in all individuals with HTN. Risk is highest in those aTRH regardless of blood pressure control. A more aggressive approach that focuses on lifestyle and pharmacologic measures to either prevent HTN or better control HTN during earlier stages may be particularly beneficial in reducing related AF risk.

## Graphical abstract

We evaluated the association of hypertension (HTN) severity with atrial fibrillation (AF) in REasons for Geographic And Racial Differences in Stroke. Compared to normotension, the risk of developing AF is increased in all individuals with HTN regardless of BP control; however, the risk is highest in those with apparent treatment‐refractory HTN.

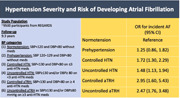

## 1 INTRODUCTION

The prevalence of atrial fibrillation (AF) has tripled over the past 50 years.^1^ It is estimated that nearly 50 million individuals are estimated to have AF worldwide, making this the most prevalent clinically significant cardiac arrhythmia.^2^ Although individuals with AF do not die directly from the arrhythmia itself, they are at a much higher risk for stroke, heart failure, and cardiovascular mortality.^3^, ^4^

Hypertension (HTN), and to a lesser extent prehypertension, are both associated with an increased risk of incident AF.^5^, ^6^, ^7^, ^8^ HTN is perhaps the most important modifiable risk factor for the development of AF, with a population attributable fraction that is estimated to be >20%.^5^, ^6^, ^7^ In longitudinal analyses looking at HTN trajectories over time, persistently elevated blood pressures as well as a greater duration of treated HTN were associated with the highest risks of developing AF.^9^, ^10^, ^11^, ^12^, ^13^

Prospective studies looking at AF risk across the spectrum of clinically defined stages (prehypertension, HTN, controlled HTN, uncontrolled HTN, and apparent treatment‐resistant hypertension [aTRH]) that concomitantly take blood pressure and medication use into account are lacking. A previous study in the REasons for Geographic And Racial Differences in Stroke (REGARDS) found no differences in AF prevalence across worsening HTN stages; however, this study was cross‐sectional.^14^ We determined whether increasing HTN severity and poorer BP control stratified according to these easily defined clinical categories are associated with a higher risk of incident AF in the REGARDS study, a large biracial prospective cohort study of men and women. Findings from the study will provide important clinical insight into how risk of developing AF may differ according to severity and control of HTN.

## 2 METHODS

Details of the methods of the REGARDS study have been published.^15^ Briefly, REGARDS is a prospective cohort study designed to identify contributors to regional and Black–White disparities in stroke mortality. The study over‐sampled Black persons and residents of the stroke belt (North Carolina, South Carolina, Georgia, Alabama, Mississippi, Tennessee, Arkansas, and Louisiana). Between January 2003 and October 2007, using postal mailings and telephone interviews, a total of 30 239 participants were recruited from a commercially available list of residents. Sociodemographic information and medical histories were obtained by a computer‐assisted telephone interview (CATI). An in‐home examination was performed 3–4 weeks after the telephone interview. Trained staff collected medication information, blood and urine samples, blood pressure readings, and a resting electrocardiogram (ECG). Approximately 10 years after the baseline assessment, 2013–2016, participants who were still alive and active completed a follow‐up examination similar to the baseline visit. The institutional review boards at the collaborating centers approved the REGARDS study protocol, and all participants provided written informed consent. Eligible participants will be those from the REGARDS cohort with baseline data on blood pressure and antihypertensive use as well as follow‐up data from the second in‐home visit for the development of AF.

### 2.1 Blood pressure measurements

BP was taken by trained examiners using android sphygmomanometer. BP was measured twice following a standard protocol. All participants were asked to sit for 5 minutes with feet on floor before BP measurement, and there was a 30‐second interval between measurements. The average of two readings was calculated. BP quality was monitored by central examination of digit preference and retraining of personnel as needed. Antihypertensive medication use was self‐reported. Medication adherence was assessed using a four‐item validated scale.^16^ HTN duration was determined based on the self‐reported history of short, medium, and long‐term (i.e., <10, 10–20, and >20 years, respectively) at the baseline visit.

### 2.2 Definition of groups based on BP and antihypertensive treatment

We will stratify the cohort into six mutually exclusive groups based on BP control and number of antihypertensive medications used.^17^ We define (1) *normotension* as SBP < 120 mmHg and DBP < 80 mmHg without antihypertensive medication use, (2) *prehypertension* as SBP 120–129 mmHg and DBP < 80 mmHg without antihypertensive medication use, (3) *controlled HTN* as SBP < 130 mmHg and DBP < 80 mmHg on ≤3 classes of antihypertensive medications, (4) *uncontrolled HTN* as SBP ≥ 130 mmHg, and/or DBP ≥ 80 mmHg on none or <3 classes of antihypertensive medications, (5) *controlled aTRH* as SBP < 130 mmHg and DBP < 80 mmHg on ≥4 classes of antihypertensive medications, and (6) *uncontrolled aTRH* as SBP ≥ 130 mmHg and/or DBP ≥ 80 mmHg on ≥3 classes of antihypertensive medications.

### 2.3 AF

AF was identified at baseline and a subsequent follow‐up visit approximately 10 years later by (1) scheduled ECG and (2) self‐reported history of a physician diagnosis during the CATI survey. The ECGs were read and coded at a central reading center (EPICARE, Wake Forest School of Medicine) by analysts who were blinded to other REGARDS data. Self‐reported AF was defined as an affirmative response to the following question: “Has a physician or a health professional ever told you that you had atrial fibrillation?” This question has been shown to be a reliable predictor of incident stroke events as AF detected by ECG.^18^

### 2.4 Covariates

Participant characteristics at baseline were used as covariates. Age, sex, race, household income, education, and smoking status were self‐reported. Body mass index (BMI) was measured at the baseline examination. Physically active was defined as ≥4 days of exercise (enough to work up a sweat) per week. Diabetes mellitus was defined as fasting glucose ≥126 mg/dL, nonfasting glucose ≥200 mg/dL, or self‐reported current use of antidiabetic medications. C‐reactive protein (CRP) measurement used a high‐sensitivity particle‐enhanced immunonephelometric assay on the BNIII nephelometer (N High‐Sensitivity CRP, Dade Behring Inc.) with an interassay coefficient of variation of 2%–6%. Using isotope‐dilution mass spectrometry traceable serum creatinine, estimated glomerular filtration rate (eGFR) was calculated using the abbreviated Modification of Diet in Renal Disease study equation.^19^ Cardiovascular disease included the presence of coronary heart disease (CHD), defined as a self‐reported history of myocardial infarction, coronary artery bypass grafting, coronary angioplasty or stenting, or evidence of prior myocardial infarction on the baseline ECG, or prior stroke which was ascertained by participant's self‐report at the time of study enrollment.

### 2.5 Statistical analysis

Participants with missing baseline exposure (blood pressure or antihypertensive use) or covariate measures, poor quality ECG recordings, baseline AF, or missing. AF follow‐up data were excluded. Descriptive statistics for demographic, socioeconomic, lifestyle, anthropometric, and medical history variables at the baseline assessment according to blood pressure groups were calculated using the *χ* ^2^ test (for proportions) and analysis of variance (for continuous variables).

Odds ratios (ORs) for incident AF were calculated among blood pressure groups (referent = normotensive). We also performed subgroup analyses to evaluate the effect modification by age (<mean vs. ≥mean), race (white vs. black), and gender (female vs. male). Multivariable logistic regression was used to compute OR and 95% confidence intervals (CI) for associations involving blood pressure categories and incident AF, overall and stratified by age, race, gender, and geographic region. Multivariable models were adjusted for as follows: Model 1 adjusted for age, sex, height, race, education, income, and geographic region. Model 2 included covariates in Model 1 + BMI, diabetes, total cholesterol, high‐density lipoprotein (HDL), eGFR, smoking, exercise, alcohol use, log‐transformed CRP, CHD, and stroke. We additionally adjusted duration of HTN and medication adherence to determine whether these factors attenuated associations. We excluded an additional 107 participants for analyses adjusting for HTN duration due to missing data. Statistical significance for all comparisons including interactions was defined as *p* < .05. SAS version 9.4 was used for all analyses.

## 3 RESULTS

**Figure 1:**
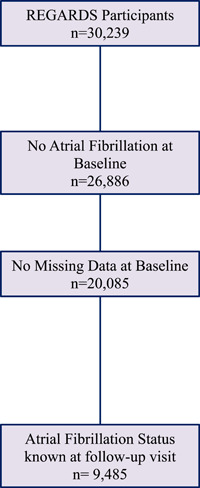
Flowchart of study participants.

Figure 1 shows the flowchart of participants. There were 20 805 REGARDS participants with complete covariate data and without AF at baseline and 9485 of these were included in the prospective analyses. Baseline characteristics stratified by blood pressure groups are shown in Table 1. Mean (SD) age was 63.3 (8.3) years, 35% were Black, and 56% female. A total of 868 (9.1%) participants developed AF over a median (interquartile range [IQR]) follow‐up of 9.3 (1.4) years. Compared to normotensive participants those with worsening HTN severity were older, more likely to be male, be black, smoke, to have diabetes, CHD, a prior stroke, and less likely to have a high school education, have an annual income >$35 000, exercise, or drink alcohol (Table 1). Kidney function and HDL‐c were lower while high‐sensitivity CRP was higher across worsening HTN severity groups.

**Table 1:** Baseline characteristics according to blood pressure groups.

Characteristics	Normotensive	Prehypertension	Controlled HTN	Uncontrolled HTN	Controlled aTRH	Uncontrolled aTRH
	*N* = 1629	*N* = 704	*N* = 2224	*N* = 4123	*N* = 88	*N* = 717
Age, mean ± SD	60.1 ± 8	62.8 ± 8.1	64.4 ± 8.4	63.5 ± 8.2	65.5 ± 7.8	65.5 ± 7.5
Black, *n* (%)	291 (17.9)	144 (20.5)	783 (35.2)	1610 (39.0)	43 (48.9)	409 (57.0)
Male, *n* (%)	585 (35.9)	335 (47.6)	914 (41.1)	1961 (47.6)	49 (55.7)	341 (47.6)
Stroke region, *n* (%)						
Nonbelt	714 (43.8)	306 (43.5)	910 (40.9)	1857 (45.0)	40 (45.5)	314 (43.8)
Belt	537 (33.0)	255 (36.2)	780 (35.1)	1410 (34.2)	28 (31.8)	248 (34.6)
Buckle	378 (23.2)	143 (20.3)	534 (24.0)	856 (20.8)	20 (22.7)	155 (21.6)
Annual household income, *n* (%)						
≥$35 000	1076 (66.1)	434 (61.6)	1184 (53.2)	2212 (53.7)	51 (58.0)	324 (45.2)
<$35 000	375 (23.0)	197 (28.0)	796 (35.8)	1501 (36.4)	29 (33.0)	319 (44.5)
Declined to report	178 (10.9)	73 (10.4)	244 (11.0)	410 (9.9)	8 (9.1)	74 (10.3)
Education <12 years, *n* (%)	43 (2.6)	31 (4.4)	171 (7.7)	346 (8.4)	3 (3.4)	81 (11.3)
BMI (kg/m^2^), mean ± SD	26 ± 4.5	27.5 ± 4.4	29.6 ± 5.7	30 ± 5.9	31.7 ± 5.7	32.5 ± 6.7
Smoking, *n* (%)						
Current	197 (12.1)	72 (10.2)	219 (9.8)	454 (11.0)	6 (6.8)	60 (8.4)
Former	541 (33.2)	290 (41.2)	922 (41.5)	1691 (41.0)	44 (50.0)	301 (42.0)
Never	891 (54.7)	342 (48.6)	1083 (48.7)	1978 (48.0)	38 (43.2)	356 (49.7)
Any exercise, *n* (%)	1219 (74.8)	549 (78.0)	1559 (70.1)	2887 (70.0)	64 (72.7)	473 (66.0)
Alcohol use, *n* (%)	839 (51.5)	340 (48.3)	863 (38.8)	1743 (42.3)	36 (40.9)	253 (35.3)
Diabetes, *n* (%)	80 (4.9)	51 (7.2)	499 (22.4)	669 (16.2)	27 (30.7)	252 (35.1)
CHD, *n* (%)	76 (4.7)	40 (5.7)	400 (18.0)	487 (11.8)	26 (29.5)	160 (22.3)
Stroke at baseline, *n* (%)	19 (1.2)	10 (1.4)	98 (4.4)	141 (3.4)	15 (17.0)	55 (7.7)
eGFR (mL/min), mean ± SD	91.1 ± 13.8	89.8 ± 14.2	84.3 ± 18.1	88.4 ± 17.3	72.9 ± 24	82.1 ± 21.3
TC (mg/dL), mean ± SD	197.8 ± 36.6	195.1 ± 35	184.8 ± 37.6	194.8 ± 38.5	173.9 ± 41	182 ± 37.3
HDL‐c (mg/dL), mean ± SD	57.2 ± 17	53.2 ± 15.5	50.8 ± 15.3	51.9 ± 15.7	47.8 ± 15.8	49.6 ± 14.7
hs‐CRP (mL/min), mean ± SD	2.8 ± 5.9	3.1 ± 5.5	4.4 ± 7.9	4.1 ± 5.6	4.3 ± 6.8	4.7 ± 7.5
Antihypertensive medication use, *n* (%)						
Overall			2224 (100.0)	2538 (61.6)	88 (100.0)	717 (100.0)
Beta‐blockers			713 (32.1)	626 (15.2)	87 (98.9)	447 (62.3)
Calcium‐channel blockers			555 (25.0)	633 (15.4)	79 (89.8)	488 (68.1)
ACEI/ARBs			1251 (56.3)	1323 (32.1)	83 (94.3)	647 (90.2)
Mineralocorticoid receptor antagonists			31 (1.4)	6 (0.1)	11 (12.5)	23 (3.2)
Diuretics			1055 (47.4)	1021 (24.8)	76 (86.4)	597 (83.3)
Other^a^			160 (7.2)	111 (2.7)	31 (35.2)	128 (17.9)

*Note*: Continuous variables are expressed as mean (SD). Categorical variables are *N* (percent).

Abbreviations: ACEI/ARBS, angiotensin‐converting enzyme inhibitor/angiotensin receptor blockers; aTRH, apparent treatment‐resistant hypertension; BMI, body mass index; CHD, coronary heart disease; eGFR, estimated glomerular filtration rate; HDL‐c, high‐density lipoprotein cholesterol; hs‐CRP, high‐sensitivity C‐reactive protein; HTN, hypertension; SD, standard deviation; TC, total cholesterol.

^a^ Other is defined by the use of alpha‐blockers, direct vasodilators, or central acting agents.

Risk of incident AF according to blood pressure groups and is reported in Table 2, respectively. In fully adjusted analyses (Model 2), participants with HTN had a higher risk of incident AF compared to those with normal blood pressure. The risk was nominally highest for those on more than three antihypertensive drugs with either controlled (OR 2.95; 95% CI 1.60, 5.43) or uncontrolled (OR 2.46; 95% CI 1.76, 3.48) blood pressure. The increase in AF risk was less for those on no more than three antihypertensive agents regardless of their blood pressure control (controlled OR 1.72; 95% CI 1.30, 2.29, and uncontrolled OR 1.48; 95% CI 1.13, 1.94). Results were not significantly changed after additional adjustment for either duration of HTN (Model 3) or medication adherence (Model 4).

**Table 2:** Risk of incident AF across blood pressure groups.

	Blood pressure groups						
	Normotensive	Prehypertension	Controlled HTN	Uncontrolled HTN	Controlled aTRH	Uncontrolled aTRH	*p‐*trend
AF (*n*)	76	51	246	364	19	112	
Total (*n*)	1629	704	2224	4123	88	717	
Model 1	REF	1.33 (0.91, 1.92)	2.25 (1.71, 2.96)	1.80 (1.39, 2.34)	5.00 (2.79, 8.97)	3.73 (2.70, 5.15)	<.0001
Model 2	REF	1.25 (0.86, 1.82)	1.72 (1.30, 2.29)	1.48 (1.13, 1.94)	2.95 (1.60, 5.43)	2.47 (1.76, 3.48)	<.0001
Model 3	REF	1.25 (0.86, 1.82)	1.56 (1.14, 2.13)	1.37 (1.03, 1.83)	2.51 (1.34, 4.72)	2.09 (1.43, 3.05)	.0019
Model 4	REF	1.25 (0.86, 1.82)	1.73 (1.30, 2.29)	1.48 (1.13, 1.94)	2.96 (1.60, 5.46)	2.49 (1.77, 3.50)	<.0001

*Note*: Model 1 = adjusted for age, sex, height, race, education, income, and geographic region. Model 2 = Model 1 + BMI, diabetes, TC, HDL, eGFR, smoking, exercise, alcohol use, log‐transformed CRP, CHD, and stroke. Model 3 = Model 2 + duration of HTN. Model 4 = Model 2 + medication adherence.

Abbreviations: AF, atrial fibrillation; aTRH, apparent treatment‐resistant hypertension; BMI, body mass index; CHD, coronary heart disease; CRP, C‐reactive protein; eGFR, estimated glomerular filtration rate; HDL, high‐density lipoprotein; HTN, hypertension; TC, total cholesterol.

In subgroup analyses, associations were stronger in older participants (Table 3), female participants (Table 4), and white participants (Table 5). Adjusted incident AF risk was not significantly increased across HTN groups in younger participants (mean age < 63.2 years) or black participants.

**Table 3:** Risk of incident AF across blood pressure groups stratified by age.

	Age ≥ median (63)						
	Normotensive	Prehypertension	Controlled HTN	Uncontrolled HTN	Controlled aTRH	Uncontrolled aTRH	*p‐*trend^a^
AF (*n*)	40	36	178	251	14	91	
Total (*n*)	583	348	1252	2137	58	451	
Model 1	REF	1.46 (0.91, 2.35)	2.36 (1.64, 3.40)	1.94 (1.36, 2.76)	5.14 (2.52, 10.4)	4.55 (3.02, 6.85)	<.0001
Model 2	REF	1.36 (0.84, 2.20)	1.86 (1.27, 2.70)	1.62 (1.13, 2.32)	3.41 (1.62, 7.17)	3.18 (2.07, 4.89)	<.0001
Model 3	REF	1.36 (0.84, 2.20)	1.75 (1.16, 2.64)	1.55 (1.06, 2.26)	2.91 (1.35, 6.27)	2.71 (1.69, 4.36)	.0004
Model 4	REF	1.36 (0.84, 2.20)	1.85 (1.27, 2.70)	1.62 (1.13, 2.32)	3.40 (1.62, 7.15)	3.18 (2.07, 4.89)	<.0001

	Age < median (63)						
	Normotensive	Prehypertension	Controlled HTN	Uncontrolled HTN	Controlled aTRH	Uncontrolled aTRH	*p‐trend*
AF (*n*)	36	15	68	113	5	21	
Total (*n*)	1046	356	972	1986	30	266	
Model 1	REF	1.09 (0.58, 2.02)	2.06 (1.35, 3.16)	1.54 (1.03, 2.28)	5.12 (1.78, 14.7)	2.14 (1.19, 3.85)	.0046
Model 2	REF	1.07 (0.57, 2.00)	1.51 (0.96, 2.35)	1.25 (0.83, 1.89)	2.30 (0.73, 7.19)	1.31 (0.70, 2.44)	.3571
Model 3	REF	1.07 (0.57, 2.00)	1.29 (0.78, 2.13)	1.12 (0.71, 1.74)	2.00 (0.62, 6.43)	1.12 (0.57, 2.22)	.8685
Model 4	REF	1.07 (0.57, 2.01)	1.52 (0.97, 2.37)	1.26 (0.83, 1.90)	2.37 (0.75, 7.45)	1.32 (0.71, 2.46)	.3450

*Note*: Model 1 = adjusted for age, sex, height, race, education, income, and geographic region. Model 2 = Model 1 + BMI, diabetes, TC, HDL, eGFR, smoking, exercise, alcohol use, log‐transformed CRP, CHD, and stroke. Model 3 = Model 2 + duration of HTN. Model 4 = Model 2 + medication adherence.

Abbreviations: AF, atrial fibrillation; aTRH, apparent treatment‐resistant hypertension; BMI, body mass index; CHD, coronary heart disease; CRP, C‐reactive protein; eGFR, estimated glomerular filtration rate; HDL, high‐density lipoprotein; HTN, hypertension; TC, total cholesterol.

^a^ The *p* value displayed in this table is the result of the Type 3 Analysis of Effects hypothesis test for the blood pressure groups.

**Table 4:** Risk of incident AF across blood pressure groups stratified by gender.

	Male						
	Normotensive	Prehypertension	Controlled HTN	Uncontrolled HTN	Controlled aTRH	Uncontrolled aTRH	*p*‐trend
AF (*n*)	41	36	149	209	13	56	
Total (*n*)	585	335	914	1961	49	341	
Model 1	REF	1.51 (0.94, 2.43)	2.43 (1.67, 3.52)	1.60 (1.12, 2.29)	5.11 (2.45, 10.7)	2.99 (1.92, 4.66)	<.0001
Model 2	REF	1.46 (0.91, 2.36)	1.78 (1.21, 2.62)	1.31 (0.91, 1.88)	2.75 (1.27, 5.95)	1.93 (1.20, 3.08)	.0954
Model 3	REF	1.46 (0.90, 2.36)	1.51 (0.99, 2.30)	1.16 (0.79, 1.71)	2.20 (0.99, 4.87)	1.52 (0.91, 2.55)	.6769
Model 4	REF	1.46 (0.90, 2.36)	1.77 (1.20, 2.61)	1.31 (0.91, 1.89)	2.72 (1.25, 5.89)	1.93 (1.20, 3.09	.0934

	Female						
	Normotensive	Prehypertension	Controlled HTN	Uncontrolled HTN	Controlled aTRH	Uncontrolled aTRH	*p‐*trend
AF (*n*)	35	15	97	155	6	56	
Total (*n*)	1044	369	1310	2162	39	376	
Model 1	REF	0.98 (0.52, 1.83)	2.00 (1.33, 3.01)	2.04 (1.38, 3.00)	4.52 (1.69, 12.1)	4.60 (2.88, 7.35)	<.0001
Model 2	REF	0.91 (0.48, 1.71)	1.62 (1.06, 2.46)	1.67 (1.12, 2.50)	3.05 (1.09, 8.54)	3.09 (1.88, 5.08)	<.0001
Model 3	REF	0.91 (0.49, 1.71)	1.64 (1.02, 2.64)	1.68 (1.09, 2.58)	3.06 (1.06, 8.84)	2.92 (1.67, 5.09)	<.0001
Model 4	REF	0.92 (0.49, 1.73)	1.62 (1.06, 2.47)	1.69 (1.13, 2.52)	3.04 (1.07, 8.62)	3.16 (1.92, 5.19)	<.0001

*Note*: Model 1 = adjusted for age, sex, height, race, education, income, and geographic region. Model 2 = Model 1 + BMI, diabetes, TC, HDL, eGFR, smoking, exercise, alcohol use, log‐transformed CRP, CHD, and stroke. Model 3 = Model 2 + duration of HTN. Model 4 = Model 2 + medication adherence.

Abbreviations: AF, atrial fibrillation; aTRH, apparent treatment‐resistant hypertension; BMI, body mass index; CHD, coronary heart disease; CRP, C‐reactive protein; eGFR, estimated glomerular filtration rate; HDL, high‐density lipoprotein; HTN, hypertension; TC, total cholesterol.

**Table 5:** Risk of incident AF across blood pressure groups stratified by race.

	White						
	Normotensive	Prehypertension	Controlled HTN	Uncontrolled HTN	Controlled aTRH	Uncontrolled aTRH	*p‐*trend
AF (*n*)	65	49	205	283	14	70	
Total (*n*)	1338	560	1441	2513	45	308	
Model 1	REF	1.51 (1.02, 2.23)	2.46 (1.82, 3.31)	1.85 (1.39, 2.47)	5.91 (2.92, 12.0)	3.84 (2.63, 5.60)	<.0001
Model 2	REF	1.45 (0.97, 2.14)	1.93 (1.42, 2.63)	1.57 (1.17, 2.10)	3.52 (1.69, 7.33)	2.62 (1.76, 3.90)	<.0001
Model 3	REF	1.45 (0.98, 2.14)	1.81 (1.29, 2.55)	1.50 (1.10, 2.05)	3.18 (1.50, 6.75)	2.32 (1.50, 3.61)	.0056
Model 4	REF	1.44 (0.97, 2.14)	1.93 (1.42, 2.63)	1.56 (1.17, 2.10)	3.53 (1.69, 7.37)	2.65 (1.78, 3.96)	<.0001

	Black						
	Normotensive	Prehypertension	Controlled HTN	Uncontrolled HTN	Controlled aTRH	Uncontrolled aTRH	*p‐*trend
AF (*n*)	11	2	41	81	5	42	
Total (*n*)	291	144	783	1610	43	409	
Model 1	REF	0.33 (0.07, 1.52)	1.30 (0.65, 2.58)	1.24 (0.65, 2.37)	2.56 (0.83, 7.94)	2.50 (1.25, 4.99)	.0002
Model 2	REF	0.28 (0.06, 1.28)	0.92 (0.45, 1.87)	0.91 (0.47, 1.78)	1.29 (0.39, 4.25)	1.49 (0.71, 3.10)	.0281
Model 3	REF	0.27 (0.06, 1.24)	0.61 (0.27, 1.39)	0.65 (0.30, 1.37)	0.70 (0.19, 2.52)	0.92 (0.39, 2.17)	.2247
Model 4	REF	0.28 (0.06, 1.28)	0.91 (0.45, 1.86)	0.90 (0.46, 1.76)	1.32 (0.40, 4.34)	1.48 (0.71, 3.09)	.0281

*Note*: Model 1 = adjusted for age, sex, height, race, education, income, and geographic region. Model 2 = Model 1 + BMI, diabetes, TC, HDL, eGFR, smoking, exercise, alcohol use, log‐transformed CRP, CHD, and stroke. Model 3 = Model 2 + duration of HTN. Model 4 = Model 2 + medication adherence.

Abbreviations: AF, atrial fibrillation; aTRH, apparent treatment‐resistant hypertension; BMI, body mass index; CHD, coronary heart disease; CRP, C‐reactive protein; eGFR, estimated glomerular filtration rate; HDL, high‐density lipoprotein; HTN, hypertension; TC, total cholesterol.

## 4 DISCUSSION

Compared to those with normotension, the risk of developing AF was highest for those with aTRH. The increase in AF risk was of lower magnitude but still significant for those on no more than three antihypertensive agents. ORs were similar regardless of achieved blood pressure control. No significant associations were observed for participants with prehypertension. Additional adjustment HTN duration or antihypertensive medication adherence did not affect associations. In subgroup analyses, associations were stronger in older participants, female participants, and white participants.

The presence of HTN has already been established not only as a risk factor for AF, but perhaps the strongest modifiable risk factor. In both the Multi‐Ethnic Study of Atherosclerosis (MESA) and the Atherosclerosis Risk in Communities study, the population‐attributable fraction of AF for HTN was >20%.^5^, ^7^ Individuals with HTN represent a heterogenous population with respect to severity, control, management, and duration. Prospective studies looking at AF risk over a range of HTN stages while accounting for blood pressure control, medication use, and duration into account are lacking. Results reported our build upon prior work by discerning whether differences in AF risk exist according to these characteristics.

It is unclear whether the relation between BP and the risk of AF is linear or whether there is a threshold BP value above which the risk is definitively increased. A prior cross‐sectional study in REGARDS found that although the prevalence of AF was higher in persons with HTN compared to those without HTN, the severity of HTN was not associated with AF prevalence.^14^ It is important to note, however, that a higher prevalence ratio for AF was seen in the aTRH groups in models that did not additionally adjust for use of antihypertensive medications. In this regard, cross‐sectional findings are like prospective associations reported here.

Prospective studies and randomized trials have had mixed results. Among over 4000 Framingham Heart Study participants, the presence of HTN regardless of achieved control was most associated with incident AF risk.^20^ Similarly, in an Italian‐based cohort of nearly 2500 initially untreated subjects with essential HTN and free of clinical cardiovascular disease no association was observed between blood pressure level the incidence of AF.^21^ In a nationwide South Korean sample of over 3 million adults, however, AF risk increased with both a higher persistence as well as severity of HTN.^22^ It has also been suggested that a U‐shaped association between HTN and incident AF exists, whereby risk for an SBP > 150 mmHg was similarly increased to an SBP < 120 mmHg compared to those with treated SBP levels between 120 and 129 mmHg.^23^ This study, however, included only participants with treated HTN and did not distinguish participants according to number of antihypertensive agents used. Finally, while results from individual randomized controlled trials for treatment of HTN have suggested lower on‐treatment BP levels are associated with a reduced risk of AF,^24^, ^25^, ^26^, ^27^ a large meta‐analysis of randomized trials comparing various antihypertensive drug regimens found that differences in blood pressure variability or mean SBP were not related to risk of new AF.^28^ Our findings suggest that accounting for the number of medications as a measure of severity of HTN may better identify individuals at higher AF risk than single BP measurements alone.

Age is the strongest AF risk factor. In the MESA cohort participants 70–79 and 80–89 years of age had an over seven‐ and ninefold respective increase in AF risk compared to those 50–59 years old.^5^ While the age‐adjusted incidence of AF is higher in men compared to women, the lifetime risk is similar. Underlying AF risk factors and taller stature are thought to largely explain the higher AF incidence in men.^1^, ^29^ Despite the generally higher burden of most primary AF risk factors, incident AF rate ratios are lower in blacks compared to whites.^5^ The lifetime AF risk is one in five for blacks compared to one in three for whites.^1^ This disproportionately lower risk in blacks is most notable in the absence of cardiovascular risk factors.^30^ The impact of HTN severity and control on these associations is unclear.^31^ We found that associations of worse HTN severity were stronger in older participants but weaker in both male and white participants. Whites with controlled aTRH also had a lower adjusted baseline prevalence of AF in this cohort compared with blacks but gender and age had no impact on associations with AF prevalence based on HTN severity.^14^

Our study has multiple strengths that include the ability to evaluate the relationship between HTN and incident AF risk with adjustment for multiple HTN characteristics (severity, medication adherence, and duration) in a biracial cohort. Limitations are present as well. Covariates were assessed at baseline only. Duration of HTN relied solely on participant recall. The sensitivity to detect AF was limited to ECG detection at the follow‐up visit. The sample size for the aTRH groups were significantly smaller, leading to wider point estimates. Results for these subgroups should be interpreted within this context. Finally, as is true with any observational study, we cannot rule out residual confounding by unmeasured covariates.

In conclusion, the risk of developing AF was increased all HTN groups and became stronger with worsening HTN severity. BP control did not influence risk. Associations were stronger in older participants, female participants, and white participants. A more aggressive approach that focuses on lifestyle and pharmacologic measures to either prevent HTN or better control HTN during earlier stages may be particularly beneficial in reducing related AF risk.

## CONFLICT OF INTEREST STATEMENT

The authors declare no conflict of interest.

## Data Availability

The data that support the findings of this study are available from the corresponding author upon reasonable request.
